# Di-μ-acetato-1:2κ^2^ *O*:*O*′;2:3κ^3^ *O*:*O*,*O*′-bis­(5,5,7,12,12,14-hexa­methyl-1,4,8,11-tetra­aza­cyclo­tetra­deca­ne)-1κ^4^ *N*,3κ^4^ *N*-bis­(perchlorato-2κ^2^ *O*,*O*′)-2-sodium-1,3-dizinc perchlorate

**DOI:** 10.1107/S1600536811045363

**Published:** 2011-11-05

**Authors:** Guang-Chuan Ou, Seik Weng Ng

**Affiliations:** a Department of Biology and Chemistry, Hunan University of Science and Engineering, Yongzhou 425100, People’s Republic of China; b Department of Chemistry, University of Malaya, 50603 Kuala Lumpur, Malaysia; c Chemistry Department, King Abdulaziz University, PO Box 80203 Jeddah, Saudi Arabia

## Abstract

In the title salt, [NaZn_2_(CH_3_COO)_2_(ClO_4_)_2_(C_16_H_36_N_4_)_2_]ClO_4_, the macrocyclic ligand binds to the Zn^2+^ cations through their four amino N atoms; the Zn^2+^ cations are also each covalently bonded to an acetate ion. For one zinc atom, the acetate group is monodentate, and the geometry is a distorted ZnN_4_O trigonal bipyramid; for the other, the acetate group is anisobidentate and the geometry is a distorted ZnN_4_O_2_ octa­hedron. The two macrocycle–zinc acetate units are bridged through a diperchloratosodium unit. In the crystal, the complex cations and uncoordinated perchlorate anions are linked by N—H⋯O hydrogen bonds.

## Related literature

For a related structure, see: Hu *et al.* (1996 ▶).
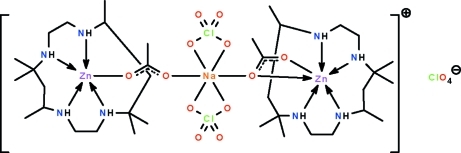


## Experimental

### 

#### Crystal data

[NaZn_2_(C_2_H_3_O_2_)_2_(ClO_4_)_2_(C_16_H_36_N_4_)_2_]ClO_4_
*M* *_r_* = 1138.14
Monoclinic, 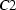

*a* = 39.151 (3) Å
*b* = 10.7100 (7) Å
*c* = 12.7446 (9) Å
β = 106.113 (2)°
*V* = 5134.0 (6) Å^3^
*Z* = 4
Mo *K*α radiation
μ = 1.17 mm^−1^
*T* = 183 K
0.45 × 0.40 × 0.15 mm

#### Data collection

Bruker SMART diffractometer
Absorption correction: multi-scan (*SADABS*; Sheldrick, 1996 ▶) *T*_min_ = 0.621, *T*_max_ = 0.844
10438 measured reflections
7701 independent reflections
5450 reflections with *I* > 2σ(*I*)
*R*_int_ = 0.038

#### Refinement

*R*[*F* ^2^ > 2σ(*F* ^2^)] = 0.062
*wR*(*F* ^2^) = 0.179
*S* = 1.12
7701 reflections
597 parameters
1 restraint
H-atom parameters constrained
Δρ_max_ = 0.81 e Å^−3^
Δρ_min_ = −0.92 e Å^−3^
Absolute structure: Flack (1983 ▶), 2251 Friedel pairs
Flack parameter: −0.07 (2)

### 

Data collection: *SMART* (Bruker, 1997 ▶); cell refinement: *SAINT* (Bruker, 2003 ▶); data reduction: *SAINT*; program(s) used to solve structure: *SHELXS97* (Sheldrick, 2008 ▶); program(s) used to refine structure: *SHELXL97* (Sheldrick, 2008 ▶); molecular graphics: *X-SEED* (Barbour, 2001 ▶); software used to prepare material for publication: *publCIF* (Westrip, 2010 ▶).

## Supplementary Material

Crystal structure: contains datablock(s) global, I. DOI: 10.1107/S1600536811045363/xu5351sup1.cif

Structure factors: contains datablock(s) I. DOI: 10.1107/S1600536811045363/xu5351Isup2.hkl

Additional supplementary materials:  crystallographic information; 3D view; checkCIF report

## Figures and Tables

**Table 1:** Hydrogen-bond geometry (Å, °)

*D*—H⋯*A*	*D*—H	H⋯*A*	*D*⋯*A*	*D*—H⋯*A*
N2—H2⋯O15^i^	0.88	2.43	3.27 (1)	160
N4—H4⋯O15^i^	0.88	2.44	3.24 (1)	152
N6—H6⋯O14^ii^	0.88	2.35	3.17 (1)	154
N8—H8⋯O15^ii^	0.88	2.46	3.29 (1)	158

Symmetry codes: (i) 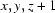; (ii) 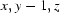.

**Fig. 1.**
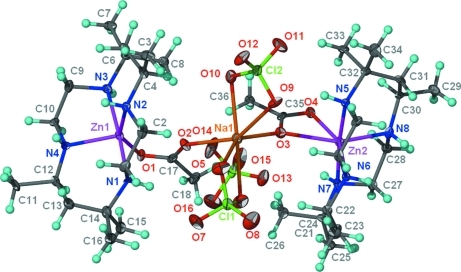
Thermal ellipsoid plot (Barbour, 2001) of [Zn2Na(ClO4)2(C2H3O)2(C16H36N4)2]+ (ClO4)- at the 30% probability level; hydrogen atoms are drawn as spheres of arbitrary radius.

## supplementary crystallographic information

### Comment

The compound was an attemped synthesis of (acetato)(*meso*-5,5,7,12,12,14-.hexamethyl-l,4,8,11-tetraazacyclotetradecane)zinc(II) perchlorate monohydrate (Hu *et al.*, 1996) but slightly different reactants were used. In the reported compound, the Zn^II^ atom is chelated by the macrocycle and is also bonded to a unidentate acetate group in a square-pyramidal geometry. The sodium cation used in the present synthesis is incorporated into the crystal structure. In the salt, [Zn_2_Na(ClO_4_)_2_(C_2_H_3_O)_2_(C_16_H_36_N_4_)_2_]^+^ (ClO_4_)^-^ (Scheme I), the *racemic* macrocyclic ligand binds to the zinc atoms through their four amino N atoms; the zinc atoms are also each covalently bonded to an acetate ion. For one zinc atom, the acetate group is unidentate, and the geometry is a trigonal bipyramid; for the other, the acetate group is anisobidentate and the geometry is an octahedron. The two macrocycle–zinc acetate units are bridged through a diperchloratosodium unit (Fig. 1). The five-coordinate geometry of the first metal atom is distorted towards a six-coordinate octahedron owing to a Zn–O contact of 2.616 (7) Å.

### Experimental

Zinc acetate dihydrate (0.183 g,1 mmol) was dissolved in 20 ml of methanol. To this solution was added [*rac*-H_2_L](ClO_4_)_2_(0.485 g, 1 mmol) (*rac*-*L*= 5,5,7,12,12,14-hexamethyl-1,4,8,11-tetraazacyclotetradecane) and sodium methoxide (0.108 g, 2 mmol). The solution was heated for an hour and then filtered. Colorless crystals were obtained after several days.

### Refinement

Carbon-bound H-atoms were placed in calculated positions (C—H 0.95 to 1.00 Å) and were included in the refinement in the riding model approximation, with *U*(H) set to 1.2 to 1.5*U*_eq_(C).

The amino H atoms were similarly treated (N–H 0.88 Å; 1.2*U*_eq_(N)).

Omitted because of bad agreement were (5 -1 1), (4 0 1), (15 -1 4), (15 1 4), (13 -3 2), (17 3 4), (15 -3 3), (16 2 4), (16 -2 4), (15 3 3), (8 0 2), (10 2 2), (10 -2 2), (24 2 7), (17 5 2), (11 1 3), (22 -4 5) and (16 4 3). The omission of these reflections led to a marginally satisfactory coverge of 95% at a 2*θ*_max_ of 50°.

### Crystal data

**Table:**

[NaZn_2_(C_2_H_3_O_2_)_2_(ClO_4_)_2_(C_16_H_36_N_4_)_2_]ClO_4_	*F*(000) = 2396
*M**_r_* = 1138.14	*D*_x_ = 1.472 Mg m^−^^3^
Monoclinic, *C*2	Mo *K*α radiation, λ = 0.71073 Å
Hall symbol: C 2y	Cell parameters from 3129 reflections
*a* = 39.151 (3) Å	θ = 2.3–23.8°
*b* = 10.7100 (7) Å	µ = 1.17 mm^−^^1^
*c* = 12.7446 (9) Å	*T* = 183 K
β = 106.113 (2)°	Block, colorless
*V* = 5134.0 (6) Å^3^	0.45 × 0.40 × 0.15 mm
*Z* = 4	

### Data collection

**Table:**

Bruker SMART diffractometer	7701 independent reflections
Radiation source: fine-focus sealed tube	5450 reflections with *I* > 2σ(*I*)
graphite	*R*_int_ = 0.038
ω scans	θ_max_ = 25.0°, θ_min_ = 1.1°
Absorption correction: multi-scan (*SADABS*; Sheldrick, 1996)	*h* = −46→26
*T*_min_ = 0.621, *T*_max_ = 0.844	*k* = −12→11
10438 measured reflections	*l* = −7→15

### Refinement

**Table:**

Refinement on *F*^2^	Secondary atom site location: difference Fourier map
Least-squares matrix: full	Hydrogen site location: inferred from neighbouring sites
*R*[*F*^2^ > 2σ(*F*^2^)] = 0.062	H-atom parameters constrained
*wR*(*F*^2^) = 0.179	*w* = 1/[σ^2^(*F*_o_^2^) + (0.0933*P*)^2^] where *P* = (*F*_o_^2^ + 2*F*_c_^2^)/3
*S* = 1.12	(Δ/σ)_max_ = 0.001
7701 reflections	Δρ_max_ = 0.81 e Å^−^^3^
597 parameters	Δρ_min_ = −0.92 e Å^−^^3^
1 restraint	Absolute structure: Flack (1983), 2251 Friedel pairs
Primary atom site location: structure-invariant direct methods	Flack parameter: −0.07 (2)

### Fractional atomic coordinates and isotropic or equivalent isotropic displacement parameters (Å^2^)

**Table:**

	*x*	*y*	*z*	*U*_iso_*/*U*_eq_	
Zn1	0.10683 (3)	0.74966 (10)	0.84956 (8)	0.0257 (3)	
Zn2	0.14679 (3)	0.19118 (10)	0.41036 (8)	0.0274 (3)	
Cl1	0.05208 (7)	0.2932 (3)	0.7007 (2)	0.0439 (7)	
Cl2	0.19253 (6)	0.2588 (2)	0.83800 (17)	0.0357 (6)	
Cl3	0.11205 (7)	0.7909 (2)	0.23602 (19)	0.0384 (6)	
Na1	0.12471 (10)	0.3799 (4)	0.6864 (3)	0.0375 (9)	
O1	0.09629 (17)	0.7798 (6)	0.6863 (5)	0.0311 (15)	
O2	0.11152 (17)	0.5821 (6)	0.7048 (5)	0.0366 (17)	
O3	0.14386 (18)	0.3468 (7)	0.5346 (5)	0.0389 (17)	
O4	0.17523 (17)	0.3652 (6)	0.4166 (5)	0.0372 (17)	
O5	0.0852 (2)	0.2939 (9)	0.7926 (7)	0.075 (3)	
O6	0.0612 (2)	0.3342 (9)	0.6053 (6)	0.066 (3)	
O7	0.0281 (3)	0.3765 (12)	0.7260 (10)	0.109 (4)	
O8	0.0375 (3)	0.1725 (10)	0.6857 (8)	0.088 (3)	
O9	0.16327 (19)	0.1873 (8)	0.7726 (5)	0.054 (2)	
O10	0.1844 (2)	0.3884 (7)	0.8145 (6)	0.058 (2)	
O11	0.2239 (2)	0.2310 (10)	0.8086 (7)	0.077 (3)	
O12	0.1982 (2)	0.2363 (9)	0.9516 (5)	0.062 (2)	
O13	0.1095 (2)	0.6609 (7)	0.2160 (7)	0.062 (2)	
O14	0.1300 (3)	0.8206 (9)	0.3420 (6)	0.093 (3)	
O15	0.1297 (3)	0.8492 (9)	0.1651 (7)	0.095 (4)	
O16	0.0782 (3)	0.8461 (12)	0.2141 (13)	0.131 (5)	
N1	0.05951 (18)	0.6399 (7)	0.8412 (6)	0.0257 (18)	
H1	0.0581	0.5835	0.7899	0.031*	
N2	0.1315 (2)	0.6344 (7)	0.9844 (6)	0.0283 (18)	
H2	0.1305	0.6757	1.0432	0.034*	
N3	0.15463 (19)	0.8610 (8)	0.8749 (6)	0.0292 (19)	
H3	0.1535	0.8941	0.8108	0.035*	
N4	0.08648 (18)	0.9064 (7)	0.9136 (6)	0.0248 (17)	
H4	0.0903	0.8918	0.9838	0.030*	
N5	0.18250 (19)	0.0971 (8)	0.5466 (6)	0.0280 (18)	
H5	0.1852	0.1484	0.6022	0.034*	
N6	0.10819 (19)	0.0707 (7)	0.4397 (6)	0.0249 (17)	
H6	0.1074	0.0052	0.3974	0.030*	
N7	0.10904 (18)	0.2693 (8)	0.2650 (5)	0.0284 (18)	
H7	0.1162	0.3464	0.2603	0.034*	
N8	0.1683 (2)	0.1013 (7)	0.2923 (6)	0.0304 (19)	
H8	0.1583	0.0274	0.2776	0.037*	
C1	0.0689 (3)	0.5712 (10)	0.9476 (8)	0.039 (3)	
H1A	0.0530	0.4982	0.9426	0.046*	
H1B	0.0656	0.6268	1.0062	0.046*	
C2	0.1072 (3)	0.5281 (10)	0.9747 (8)	0.043 (3)	
H2A	0.1132	0.4812	1.0444	0.051*	
H2B	0.1102	0.4712	0.9167	0.051*	
C3	0.1831 (3)	0.5142 (11)	1.0978 (8)	0.046 (3)	
H3A	0.1681	0.4394	1.0881	0.069*	
H3B	0.1822	0.5576	1.1647	0.069*	
H3C	0.2076	0.4901	1.1033	0.069*	
C4	0.1696 (3)	0.6002 (10)	1.0009 (8)	0.036 (3)	
H4A	0.1713	0.5543	0.9343	0.043*	
C5	0.1930 (3)	0.7165 (10)	1.0128 (8)	0.040 (3)	
H5A	0.2180	0.6893	1.0418	0.048*	
H5B	0.1875	0.7703	1.0691	0.048*	
C6	0.1908 (2)	0.7986 (10)	0.9117 (7)	0.031 (2)	
C7	0.2200 (3)	0.8946 (11)	0.9398 (9)	0.043 (3)	
H7A	0.2189	0.9462	0.8755	0.064*	
H7B	0.2430	0.8525	0.9629	0.064*	
H7C	0.2169	0.9477	0.9992	0.064*	
C8	0.1934 (3)	0.7167 (10)	0.8146 (7)	0.038 (3)	
H8A	0.1916	0.7696	0.7506	0.056*	
H8B	0.1740	0.6556	0.7980	0.056*	
H8C	0.2162	0.6728	0.8337	0.056*	
C9	0.1496 (3)	0.9672 (10)	0.9446 (8)	0.039 (3)	
H9A	0.1656	1.0369	0.9393	0.046*	
H9B	0.1552	0.9405	1.0219	0.046*	
C10	0.1114 (2)	1.0088 (9)	0.9051 (8)	0.030 (2)	
H10A	0.1076	1.0810	0.9492	0.036*	
H10B	0.1062	1.0363	0.8281	0.036*	
C11	0.0378 (3)	1.0508 (10)	0.9251 (9)	0.043 (3)	
H11A	0.0526	1.1217	0.9168	0.065*	
H11B	0.0407	1.0353	1.0029	0.065*	
H11C	0.0128	1.0696	0.8889	0.065*	
C12	0.0492 (3)	0.9325 (10)	0.8722 (9)	0.037 (3)	
H12	0.0446	0.9504	0.7924	0.045*	
C13	0.0257 (3)	0.8221 (11)	0.8822 (9)	0.046 (3)	
H13A	0.0331	0.7946	0.9594	0.055*	
H13B	0.0011	0.8540	0.8677	0.055*	
C14	0.0244 (3)	0.7078 (11)	0.8124 (8)	0.044 (3)	
C15	0.0149 (3)	0.7368 (12)	0.6918 (8)	0.050 (3)	
H15A	−0.0077	0.7823	0.6705	0.074*	
H15B	0.0126	0.6588	0.6504	0.074*	
H15C	0.0336	0.7883	0.6764	0.074*	
C16	−0.0048 (3)	0.6221 (11)	0.8330 (10)	0.052 (3)	
H16A	−0.0275	0.6674	0.8157	0.077*	
H16B	0.0017	0.5968	0.9099	0.077*	
H16C	−0.0073	0.5478	0.7866	0.077*	
C17	0.0985 (2)	0.6748 (10)	0.6461 (7)	0.032 (2)	
C18	0.0854 (3)	0.6584 (11)	0.5271 (8)	0.048 (3)	
H18A	0.0836	0.7400	0.4913	0.072*	
H18B	0.0620	0.6186	0.5087	0.072*	
H18C	0.1021	0.6055	0.5020	0.072*	
C19	0.1620 (3)	−0.0105 (10)	0.5713 (8)	0.035 (2)	
H19A	0.1632	−0.0812	0.5224	0.042*	
H19B	0.1725	−0.0380	0.6476	0.042*	
C20	0.1242 (3)	0.0269 (9)	0.5555 (7)	0.032 (2)	
H20A	0.1230	0.0948	0.6070	0.039*	
H20B	0.1105	−0.0451	0.5710	0.039*	
C21	0.0459 (3)	0.0195 (12)	0.4434 (10)	0.050 (3)	
H21A	0.0217	0.0536	0.4274	0.076*	
H21B	0.0541	−0.0033	0.5208	0.076*	
H21C	0.0458	−0.0548	0.3984	0.076*	
C22	0.0707 (2)	0.1173 (9)	0.4179 (8)	0.031 (2)	
H22	0.0708	0.1916	0.4657	0.037*	
C23	0.0572 (3)	0.1594 (9)	0.2984 (8)	0.034 (3)	
H23	0.0406	0.1089	0.2472	0.041*	
C24	0.0699 (2)	0.2805 (9)	0.2615 (7)	0.030 (2)	
C25	0.0472 (3)	0.3110 (10)	0.1480 (7)	0.037 (2)	
H25A	0.0223	0.3174	0.1484	0.055*	
H25B	0.0497	0.2448	0.0975	0.055*	
H25C	0.0550	0.3907	0.1244	0.055*	
C26	0.0673 (3)	0.3873 (10)	0.3381 (8)	0.036 (2)	
H26A	0.0424	0.3987	0.3376	0.053*	
H26B	0.0762	0.4644	0.3134	0.053*	
H26C	0.0816	0.3675	0.4123	0.053*	
C27	0.1164 (2)	0.2028 (10)	0.1725 (7)	0.035 (2)	
H27A	0.1079	0.2534	0.1052	0.042*	
H27B	0.1034	0.1224	0.1607	0.042*	
C28	0.1563 (2)	0.1789 (10)	0.1958 (7)	0.034 (2)	
H28A	0.1612	0.1367	0.1325	0.041*	
H28B	0.1692	0.2593	0.2076	0.041*	
C29	0.2221 (3)	0.0238 (12)	0.2412 (8)	0.047 (3)	
H29A	0.2155	0.0756	0.1752	0.070*	
H29B	0.2118	−0.0596	0.2244	0.070*	
H29C	0.2481	0.0170	0.2671	0.070*	
C30	0.2081 (2)	0.0837 (10)	0.3294 (7)	0.029 (2)	
H30	0.2194	0.1678	0.3463	0.035*	
C31	0.2191 (3)	0.0053 (10)	0.4327 (8)	0.034 (2)	
H31A	0.2434	−0.0264	0.4399	0.040*	
H31B	0.2032	−0.0682	0.4219	0.040*	
C32	0.2191 (2)	0.0661 (9)	0.5430 (8)	0.032 (2)	
C33	0.2399 (2)	0.1878 (11)	0.5590 (8)	0.037 (2)	
H33A	0.2403	0.2240	0.6299	0.055*	
H33B	0.2285	0.2463	0.5007	0.055*	
H33C	0.2643	0.1715	0.5567	0.055*	
C34	0.2379 (3)	−0.0269 (11)	0.6321 (8)	0.049 (3)	
H34A	0.2393	0.0084	0.7041	0.074*	
H34B	0.2619	−0.0435	0.6263	0.074*	
H34C	0.2244	−0.1051	0.6228	0.074*	
C35	0.1642 (2)	0.4109 (9)	0.4931 (8)	0.032 (2)	
C36	0.1752 (3)	0.5416 (11)	0.5329 (10)	0.048 (3)	
H36A	0.1595	0.5724	0.5753	0.073*	
H36B	0.1734	0.5966	0.4701	0.073*	
H36C	0.1998	0.5406	0.5791	0.073*	

### Atomic displacement parameters (Å^2^)

**Table:**

	*U*^11^	*U*^22^	*U*^33^	*U*^12^	*U*^13^	*U*^23^
Zn1	0.0287 (6)	0.0284 (6)	0.0229 (5)	0.0029 (5)	0.0119 (4)	0.0016 (5)
Zn2	0.0314 (6)	0.0273 (6)	0.0274 (5)	−0.0005 (5)	0.0143 (5)	0.0007 (5)
Cl1	0.0435 (16)	0.0404 (16)	0.0548 (16)	−0.0081 (13)	0.0254 (14)	−0.0079 (12)
Cl2	0.0394 (14)	0.0359 (14)	0.0314 (11)	0.0029 (12)	0.0092 (10)	−0.0040 (11)
Cl3	0.0541 (17)	0.0345 (15)	0.0340 (12)	−0.0088 (12)	0.0244 (12)	−0.0074 (11)
Na1	0.036 (2)	0.039 (2)	0.040 (2)	0.0006 (18)	0.0160 (18)	−0.0010 (18)
O1	0.047 (4)	0.024 (4)	0.025 (3)	−0.001 (3)	0.015 (3)	−0.002 (3)
O2	0.042 (4)	0.035 (4)	0.037 (4)	0.017 (3)	0.019 (3)	0.015 (3)
O3	0.037 (4)	0.038 (4)	0.046 (4)	−0.004 (3)	0.019 (3)	−0.001 (3)
O4	0.038 (4)	0.029 (4)	0.046 (4)	0.002 (3)	0.014 (3)	−0.004 (3)
O5	0.064 (6)	0.088 (8)	0.065 (5)	−0.024 (5)	0.007 (4)	0.009 (5)
O6	0.063 (6)	0.091 (7)	0.047 (5)	−0.019 (5)	0.019 (4)	−0.005 (5)
O7	0.057 (6)	0.127 (10)	0.150 (10)	0.021 (6)	0.039 (7)	−0.050 (9)
O8	0.107 (8)	0.065 (7)	0.096 (7)	−0.036 (6)	0.034 (6)	−0.001 (6)
O9	0.050 (5)	0.064 (5)	0.038 (4)	−0.020 (4)	−0.004 (3)	−0.008 (4)
O10	0.064 (5)	0.037 (5)	0.063 (5)	0.016 (4)	0.000 (4)	−0.009 (4)
O11	0.053 (5)	0.111 (9)	0.073 (5)	0.029 (5)	0.029 (4)	−0.003 (6)
O12	0.081 (6)	0.074 (6)	0.031 (4)	−0.036 (5)	0.013 (4)	−0.008 (4)
O13	0.088 (6)	0.038 (5)	0.065 (5)	−0.021 (4)	0.032 (5)	−0.011 (4)
O14	0.166 (10)	0.068 (7)	0.032 (4)	−0.005 (7)	0.005 (5)	−0.020 (4)
O15	0.175 (11)	0.069 (7)	0.071 (6)	−0.049 (7)	0.088 (7)	−0.017 (5)
O16	0.057 (7)	0.082 (9)	0.244 (16)	0.005 (6)	0.023 (9)	−0.031 (10)
N1	0.015 (4)	0.030 (5)	0.028 (4)	−0.006 (3)	−0.001 (3)	−0.011 (3)
N2	0.033 (5)	0.030 (5)	0.021 (4)	0.004 (4)	0.006 (3)	−0.001 (3)
N3	0.025 (4)	0.037 (5)	0.027 (4)	0.002 (4)	0.010 (3)	0.002 (4)
N4	0.021 (4)	0.027 (5)	0.026 (4)	−0.004 (3)	0.005 (3)	0.000 (3)
N5	0.022 (4)	0.035 (5)	0.031 (4)	−0.006 (4)	0.013 (3)	−0.008 (4)
N6	0.028 (4)	0.024 (4)	0.024 (4)	0.001 (4)	0.010 (3)	0.000 (3)
N7	0.034 (4)	0.024 (5)	0.029 (4)	0.005 (4)	0.012 (3)	0.002 (3)
N8	0.042 (5)	0.024 (4)	0.030 (4)	−0.010 (4)	0.018 (4)	−0.001 (3)
C1	0.043 (7)	0.037 (6)	0.038 (6)	−0.011 (5)	0.015 (5)	−0.002 (5)
C2	0.074 (9)	0.026 (6)	0.036 (6)	−0.007 (6)	0.028 (6)	0.003 (5)
C3	0.037 (7)	0.059 (8)	0.036 (6)	0.009 (5)	0.001 (5)	0.019 (5)
C4	0.041 (7)	0.039 (7)	0.029 (5)	0.016 (5)	0.013 (5)	0.003 (5)
C5	0.033 (6)	0.051 (8)	0.040 (5)	0.007 (5)	0.017 (4)	−0.005 (5)
C6	0.020 (5)	0.046 (7)	0.030 (5)	−0.001 (5)	0.011 (4)	−0.003 (5)
C7	0.029 (6)	0.056 (8)	0.045 (6)	−0.008 (5)	0.012 (5)	0.001 (5)
C8	0.033 (6)	0.045 (8)	0.037 (5)	−0.001 (5)	0.013 (4)	−0.004 (5)
C9	0.053 (7)	0.037 (7)	0.034 (5)	−0.010 (5)	0.024 (5)	−0.012 (5)
C10	0.021 (5)	0.028 (6)	0.042 (5)	−0.014 (4)	0.011 (4)	−0.009 (4)
C11	0.039 (6)	0.044 (7)	0.053 (7)	0.017 (5)	0.023 (5)	−0.006 (5)
C12	0.032 (6)	0.039 (6)	0.042 (6)	0.006 (5)	0.014 (5)	−0.009 (5)
C13	0.038 (6)	0.062 (8)	0.041 (6)	0.008 (6)	0.017 (5)	−0.010 (6)
C14	0.046 (7)	0.046 (7)	0.049 (6)	−0.003 (5)	0.026 (5)	−0.012 (5)
C15	0.055 (7)	0.045 (7)	0.042 (6)	0.014 (6)	0.001 (5)	−0.010 (6)
C16	0.036 (7)	0.051 (8)	0.074 (8)	−0.019 (6)	0.025 (6)	−0.020 (6)
C17	0.032 (6)	0.047 (7)	0.019 (4)	−0.011 (5)	0.008 (4)	−0.008 (5)
C18	0.054 (7)	0.054 (8)	0.029 (5)	−0.006 (6)	0.002 (5)	−0.013 (5)
C19	0.038 (6)	0.043 (7)	0.027 (5)	−0.017 (5)	0.013 (4)	−0.006 (4)
C20	0.043 (6)	0.031 (6)	0.032 (5)	−0.007 (5)	0.025 (5)	−0.002 (4)
C21	0.039 (7)	0.058 (8)	0.062 (7)	−0.014 (6)	0.027 (6)	−0.005 (6)
C22	0.030 (6)	0.027 (6)	0.039 (6)	−0.004 (5)	0.016 (5)	0.003 (4)
C23	0.040 (6)	0.035 (7)	0.029 (5)	−0.008 (5)	0.010 (5)	−0.007 (4)
C24	0.024 (5)	0.031 (6)	0.028 (5)	0.012 (4)	−0.002 (4)	0.008 (4)
C25	0.035 (6)	0.044 (7)	0.026 (5)	0.004 (5)	0.000 (4)	0.000 (5)
C26	0.042 (6)	0.033 (6)	0.032 (5)	0.000 (5)	0.008 (5)	0.000 (5)
C27	0.039 (6)	0.041 (6)	0.023 (4)	0.010 (5)	0.005 (4)	−0.001 (5)
C28	0.027 (5)	0.046 (7)	0.032 (5)	−0.002 (5)	0.014 (4)	−0.001 (5)
C29	0.033 (6)	0.078 (9)	0.036 (6)	0.011 (6)	0.018 (5)	−0.007 (6)
C30	0.019 (5)	0.037 (6)	0.029 (5)	−0.001 (4)	0.000 (4)	−0.009 (4)
C31	0.023 (6)	0.035 (6)	0.043 (6)	0.011 (4)	0.010 (5)	0.008 (5)
C32	0.024 (5)	0.036 (6)	0.038 (5)	−0.007 (4)	0.012 (4)	−0.007 (5)
C33	0.017 (5)	0.054 (7)	0.041 (5)	−0.006 (5)	0.010 (4)	−0.009 (6)
C34	0.059 (8)	0.060 (8)	0.034 (6)	0.006 (6)	0.021 (5)	0.003 (5)
C35	0.025 (6)	0.033 (6)	0.036 (5)	−0.014 (5)	0.006 (5)	−0.012 (5)
C36	0.036 (7)	0.045 (7)	0.067 (8)	−0.001 (5)	0.020 (6)	−0.016 (6)

### Geometric parameters (Å, °)

**Table:**

Zn1—O1	2.032 (6)	C8—H8A	0.9800
Zn1—N4	2.116 (7)	C8—H8B	0.9800
Zn1—N2	2.118 (7)	C8—H8C	0.9800
Zn1—N3	2.166 (8)	C9—C10	1.506 (14)
Zn1—N1	2.172 (7)	C9—H9A	0.9900
Zn1—O2	2.616 (7)	C9—H9B	0.9900
Zn2—N6	2.099 (7)	C10—H10A	0.9900
Zn2—N8	2.145 (7)	C10—H10B	0.9900
Zn2—N5	2.153 (8)	C11—C12	1.559 (14)
Zn2—O4	2.161 (7)	C11—H11A	0.9800
Zn2—N7	2.190 (7)	C11—H11B	0.9800
Zn2—O3	2.323 (7)	C11—H11C	0.9800
Cl1—O7	1.396 (10)	C12—C13	1.525 (15)
Cl1—O8	1.405 (10)	C12—H12	1.0000
Cl1—O6	1.428 (8)	C13—C14	1.506 (15)
Cl1—O5	1.486 (9)	C13—H13A	0.9900
Cl2—O11	1.411 (7)	C13—H13B	0.9900
Cl2—O12	1.422 (7)	C14—C15	1.509 (14)
Cl2—O9	1.437 (7)	C14—C16	1.545 (14)
Cl2—O10	1.437 (8)	C15—H15A	0.9800
Cl3—O14	1.376 (8)	C15—H15B	0.9800
Cl3—O16	1.407 (11)	C15—H15C	0.9800
Cl3—O13	1.414 (8)	C16—H16A	0.9800
Cl3—O15	1.426 (8)	C16—H16B	0.9800
Na1—O2	2.254 (8)	C16—H16C	0.9800
Na1—O3	2.288 (7)	C17—C18	1.471 (12)
Na1—O10	2.452 (9)	C18—H18A	0.9800
Na1—O6	2.462 (9)	C18—H18B	0.9800
Na1—O5	2.498 (9)	C18—H18C	0.9800
Na1—O9	2.612 (9)	C19—C20	1.490 (14)
O1—C17	1.248 (11)	C19—H19A	0.9900
O2—C17	1.262 (12)	C19—H19B	0.9900
O3—C35	1.272 (11)	C20—H20A	0.9900
O4—C35	1.269 (11)	C20—H20B	0.9900
N1—C1	1.496 (12)	C21—C22	1.524 (14)
N1—C14	1.509 (13)	C21—H21A	0.9800
N1—H1	0.8800	C21—H21B	0.9800
N2—C2	1.466 (13)	C21—H21C	0.9800
N2—C4	1.492 (12)	C22—C23	1.535 (13)
N2—H2	0.8800	C22—H22	1.0000
N3—C9	1.490 (12)	C23—C24	1.511 (14)
N3—C6	1.516 (12)	C23—H23	0.9500
N3—H3	0.8800	C24—C25	1.508 (12)
N4—C12	1.433 (12)	C24—C26	1.526 (13)
N4—C10	1.491 (11)	C25—H25A	0.9800
N4—H4	0.8800	C25—H25B	0.9800
N5—C32	1.484 (11)	C25—H25C	0.9800
N5—C19	1.487 (12)	C26—H26A	0.9800
N5—H5	0.8800	C26—H26B	0.9800
N6—C22	1.503 (12)	C26—H26C	0.9800
N6—C20	1.509 (11)	C27—C28	1.528 (12)
N6—H6	0.8800	C27—H27A	0.9900
N7—C27	1.472 (11)	C27—H27B	0.9900
N7—C24	1.524 (11)	C28—H28A	0.9900
N7—H7	0.8800	C28—H28B	0.9900
N8—C28	1.451 (12)	C29—C30	1.522 (12)
N8—C30	1.512 (12)	C29—H29A	0.9800
N8—H8	0.8800	C29—H29B	0.9800
C1—C2	1.515 (15)	C29—H29C	0.9800
C1—H1A	0.9900	C30—C31	1.519 (13)
C1—H1B	0.9900	C30—H30	1.0000
C2—H2A	0.9900	C31—C32	1.550 (13)
C2—H2B	0.9900	C31—H31A	0.9900
C3—C4	1.513 (13)	C31—H31B	0.9900
C3—H3A	0.9800	C32—C33	1.519 (14)
C3—H3B	0.9800	C32—C34	1.536 (14)
C3—H3C	0.9800	C33—H33A	0.9800
C4—C5	1.529 (15)	C33—H33B	0.9800
C4—H4A	1.0000	C33—H33C	0.9800
C5—C6	1.542 (14)	C34—H34A	0.9800
C5—H5A	0.9900	C34—H34B	0.9800
C5—H5B	0.9900	C34—H34C	0.9800
C6—C7	1.505 (14)	C35—C36	1.510 (15)
C6—C8	1.543 (13)	C36—H36A	0.9800
C7—H7A	0.9800	C36—H36B	0.9800
C7—H7B	0.9800	C36—H36C	0.9800
C7—H7C	0.9800		
			
O1—Zn1—N4	106.1 (3)	H8A—C8—H8C	109.5
O1—Zn1—N2	147.2 (3)	H8B—C8—H8C	109.5
N4—Zn1—N2	106.6 (3)	N3—C9—C10	108.1 (8)
O1—Zn1—N3	89.2 (3)	N3—C9—H9A	110.1
N4—Zn1—N3	84.7 (3)	C10—C9—H9A	110.1
N2—Zn1—N3	91.1 (3)	N3—C9—H9B	110.1
O1—Zn1—N1	96.2 (3)	C10—C9—H9B	110.1
N4—Zn1—N1	92.4 (3)	H9A—C9—H9B	108.4
N2—Zn1—N1	85.1 (3)	N4—C10—C9	111.5 (8)
N3—Zn1—N1	174.4 (3)	N4—C10—H10A	109.3
N6—Zn2—N8	106.9 (3)	C9—C10—H10A	109.3
N6—Zn2—N5	84.7 (3)	N4—C10—H10B	109.3
N8—Zn2—N5	93.6 (3)	C9—C10—H10B	109.3
N6—Zn2—O4	155.9 (3)	H10A—C10—H10B	108.0
N8—Zn2—O4	96.6 (3)	C12—C11—H11A	109.5
N5—Zn2—O4	99.3 (3)	C12—C11—H11B	109.5
N6—Zn2—N7	92.0 (3)	H11A—C11—H11B	109.5
N8—Zn2—N7	83.2 (3)	C12—C11—H11C	109.5
N5—Zn2—N7	174.5 (3)	H11A—C11—H11C	109.5
O4—Zn2—N7	85.5 (3)	H11B—C11—H11C	109.5
N6—Zn2—O3	98.4 (3)	N4—C12—C13	113.2 (9)
N8—Zn2—O3	154.6 (3)	N4—C12—C11	112.6 (8)
N5—Zn2—O3	86.0 (3)	C13—C12—C11	109.9 (8)
O4—Zn2—O3	58.6 (2)	N4—C12—H12	106.9
N7—Zn2—O3	98.8 (3)	C13—C12—H12	106.9
O7—Cl1—O8	110.2 (7)	C11—C12—H12	106.9
O7—Cl1—O6	110.1 (7)	C14—C13—C12	120.2 (8)
O8—Cl1—O6	110.9 (6)	C14—C13—H13A	107.3
O7—Cl1—O5	108.1 (6)	C12—C13—H13A	107.3
O8—Cl1—O5	110.2 (6)	C14—C13—H13B	107.3
O6—Cl1—O5	107.4 (5)	C12—C13—H13B	107.3
O11—Cl2—O12	109.4 (5)	H13A—C13—H13B	106.9
O11—Cl2—O9	110.2 (5)	C13—C14—N1	111.6 (8)
O12—Cl2—O9	112.0 (5)	C13—C14—C15	113.1 (10)
O11—Cl2—O10	107.9 (6)	N1—C14—C15	107.3 (7)
O12—Cl2—O10	109.8 (5)	C13—C14—C16	106.8 (8)
O9—Cl2—O10	107.4 (5)	N1—C14—C16	109.9 (9)
O14—Cl3—O16	107.1 (8)	C15—C14—C16	108.1 (9)
O14—Cl3—O13	113.3 (6)	C14—C15—H15A	109.5
O16—Cl3—O13	111.2 (6)	C14—C15—H15B	109.5
O14—Cl3—O15	108.4 (6)	H15A—C15—H15B	109.5
O16—Cl3—O15	106.7 (8)	C14—C15—H15C	109.5
O13—Cl3—O15	109.9 (5)	H15A—C15—H15C	109.5
O2—Na1—O3	112.0 (3)	H15B—C15—H15C	109.5
O2—Na1—O10	95.6 (3)	C14—C16—H16A	109.5
O3—Na1—O10	95.4 (3)	C14—C16—H16B	109.5
O2—Na1—O6	90.0 (3)	H16A—C16—H16B	109.5
O3—Na1—O6	98.3 (3)	C14—C16—H16C	109.5
O10—Na1—O6	162.0 (3)	H16A—C16—H16C	109.5
O2—Na1—O5	95.9 (3)	H16B—C16—H16C	109.5
O3—Na1—O5	143.0 (3)	O1—C17—O2	121.9 (8)
O10—Na1—O5	105.8 (3)	O1—C17—C18	119.1 (10)
O6—Na1—O5	56.5 (3)	O2—C17—C18	119.0 (10)
O2—Na1—O9	146.9 (3)	C17—C18—H18A	109.5
O3—Na1—O9	86.9 (3)	C17—C18—H18B	109.5
O10—Na1—O9	54.3 (2)	H18A—C18—H18B	109.5
O6—Na1—O9	114.7 (3)	C17—C18—H18C	109.5
O5—Na1—O9	81.6 (3)	H18A—C18—H18C	109.5
C17—O1—Zn1	105.0 (6)	H18B—C18—H18C	109.5
C17—O2—Na1	139.5 (6)	C20—C19—N5	109.7 (8)
C35—O3—Na1	131.1 (6)	C20—C19—H19A	109.7
C35—O3—Zn2	87.1 (5)	N5—C19—H19A	109.7
Na1—O3—Zn2	141.0 (3)	C20—C19—H19B	109.7
C35—O4—Zn2	94.5 (6)	N5—C19—H19B	109.7
Cl1—O5—Na1	96.4 (4)	H19A—C19—H19B	108.2
Cl1—O6—Na1	99.6 (4)	C19—C20—N6	110.1 (7)
Cl2—O9—Na1	95.6 (4)	C19—C20—H20A	109.6
Cl2—O10—Na1	102.6 (4)	N6—C20—H20A	109.6
C1—N1—C14	115.8 (7)	C19—C20—H20B	109.6
C1—N1—Zn1	103.3 (5)	N6—C20—H20B	109.6
C14—N1—Zn1	117.3 (6)	H20A—C20—H20B	108.2
C1—N1—H1	106.6	C22—C21—H21A	109.5
C14—N1—H1	106.6	C22—C21—H21B	109.5
Zn1—N1—H1	106.6	H21A—C21—H21B	109.5
C2—N2—C4	114.8 (8)	C22—C21—H21C	109.5
C2—N2—Zn1	104.2 (6)	H21A—C21—H21C	109.5
C4—N2—Zn1	118.0 (5)	H21B—C21—H21C	109.5
C2—N2—H2	106.3	N6—C22—C21	112.3 (8)
C4—N2—H2	106.3	N6—C22—C23	109.3 (7)
Zn1—N2—H2	106.3	C21—C22—C23	110.4 (8)
C9—N3—C6	114.8 (7)	N6—C22—H22	108.2
C9—N3—Zn1	104.9 (6)	C21—C22—H22	108.2
C6—N3—Zn1	119.8 (6)	C23—C22—H22	108.2
C9—N3—H3	105.4	C24—C23—C22	120.5 (8)
C6—N3—H3	105.4	C24—C23—H23	119.7
Zn1—N3—H3	105.4	C22—C23—H23	119.7
C12—N4—C10	116.7 (8)	C25—C24—C23	109.1 (8)
C12—N4—Zn1	117.7 (6)	C25—C24—C26	108.9 (8)
C10—N4—Zn1	103.4 (5)	C23—C24—C26	110.9 (7)
C12—N4—H4	106.0	C25—C24—N7	111.4 (7)
C10—N4—H4	106.0	C23—C24—N7	109.9 (7)
Zn1—N4—H4	106.0	C26—C24—N7	106.7 (7)
C32—N5—C19	114.6 (8)	C24—C25—H25A	109.5
C32—N5—Zn2	119.8 (5)	C24—C25—H25B	109.5
C19—N5—Zn2	105.4 (6)	H25A—C25—H25B	109.5
C32—N5—H5	105.3	C24—C25—H25C	109.5
C19—N5—H5	105.3	H25A—C25—H25C	109.5
Zn2—N5—H5	105.3	H25B—C25—H25C	109.5
C22—N6—C20	113.9 (6)	C24—C26—H26A	109.5
C22—N6—Zn2	118.6 (5)	C24—C26—H26B	109.5
C20—N6—Zn2	103.9 (5)	H26A—C26—H26B	109.5
C22—N6—H6	106.6	C24—C26—H26C	109.5
C20—N6—H6	106.6	H26A—C26—H26C	109.5
Zn2—N6—H6	106.6	H26B—C26—H26C	109.5
C27—N7—C24	115.6 (7)	N7—C27—C28	109.9 (7)
C27—N7—Zn2	104.9 (5)	N7—C27—H27A	109.7
C24—N7—Zn2	119.4 (5)	C28—C27—H27A	109.7
C27—N7—H7	105.2	N7—C27—H27B	109.7
C24—N7—H7	105.2	C28—C27—H27B	109.7
Zn2—N7—H7	105.2	H27A—C27—H27B	108.2
C28—N8—C30	113.6 (7)	N8—C28—C27	109.6 (7)
C28—N8—Zn2	103.8 (6)	N8—C28—H28A	109.8
C30—N8—Zn2	114.1 (5)	C27—C28—H28A	109.8
C28—N8—H8	108.3	N8—C28—H28B	109.8
C30—N8—H8	108.3	C27—C28—H28B	109.8
Zn2—N8—H8	108.3	H28A—C28—H28B	108.2
N1—C1—C2	109.5 (8)	C30—C29—H29A	109.5
N1—C1—H1A	109.8	C30—C29—H29B	109.5
C2—C1—H1A	109.8	H29A—C29—H29B	109.5
N1—C1—H1B	109.8	C30—C29—H29C	109.5
C2—C1—H1B	109.8	H29A—C29—H29C	109.5
H1A—C1—H1B	108.2	H29B—C29—H29C	109.5
N2—C2—C1	111.1 (8)	N8—C30—C29	111.9 (7)
N2—C2—H2A	109.4	N8—C30—C31	111.0 (7)
C1—C2—H2A	109.4	C29—C30—C31	109.5 (8)
N2—C2—H2B	109.4	N8—C30—H30	108.1
C1—C2—H2B	109.4	C29—C30—H30	108.1
H2A—C2—H2B	108.0	C31—C30—H30	108.1
C4—C3—H3A	109.5	C30—C31—C32	118.9 (8)
C4—C3—H3B	109.5	C30—C31—H31A	107.6
H3A—C3—H3B	109.5	C32—C31—H31A	107.6
C4—C3—H3C	109.5	C30—C31—H31B	107.6
H3A—C3—H3C	109.5	C32—C31—H31B	107.6
H3B—C3—H3C	109.5	H31A—C31—H31B	107.0
N2—C4—C3	111.9 (8)	N5—C32—C33	106.8 (8)
N2—C4—C5	111.2 (8)	N5—C32—C34	113.0 (8)
C3—C4—C5	110.5 (9)	C33—C32—C34	109.2 (8)
N2—C4—H4A	107.7	N5—C32—C31	111.4 (7)
C3—C4—H4A	107.7	C33—C32—C31	110.3 (8)
C5—C4—H4A	107.7	C34—C32—C31	106.1 (8)
C4—C5—C6	119.2 (8)	C32—C33—H33A	109.5
C4—C5—H5A	107.5	C32—C33—H33B	109.5
C6—C5—H5A	107.5	H33A—C33—H33B	109.5
C4—C5—H5B	107.5	C32—C33—H33C	109.5
C6—C5—H5B	107.5	H33A—C33—H33C	109.5
H5A—C5—H5B	107.0	H33B—C33—H33C	109.5
C7—C6—N3	110.7 (9)	C32—C34—H34A	109.5
C7—C6—C8	111.3 (8)	C32—C34—H34B	109.5
N3—C6—C8	105.5 (7)	H34A—C34—H34B	109.5
C7—C6—C5	109.1 (8)	C32—C34—H34C	109.5
N3—C6—C5	109.9 (7)	H34A—C34—H34C	109.5
C8—C6—C5	110.2 (8)	H34B—C34—H34C	109.5
C6—C7—H7A	109.5	O4—C35—O3	119.8 (9)
C6—C7—H7B	109.5	O4—C35—C36	119.3 (9)
H7A—C7—H7B	109.5	O3—C35—C36	120.9 (9)
C6—C7—H7C	109.5	C35—C36—H36A	109.5
H7A—C7—H7C	109.5	C35—C36—H36B	109.5
H7B—C7—H7C	109.5	H36A—C36—H36B	109.5
C6—C8—H8A	109.5	C35—C36—H36C	109.5
C6—C8—H8B	109.5	H36A—C36—H36C	109.5
H8A—C8—H8B	109.5	H36B—C36—H36C	109.5
C6—C8—H8C	109.5		
			
N4—Zn1—O1—C17	−160.9 (6)	N7—Zn2—N6—C20	−165.1 (5)
N2—Zn1—O1—C17	23.9 (9)	O3—Zn2—N6—C20	−65.9 (5)
N3—Zn1—O1—C17	114.8 (6)	N6—Zn2—N7—C27	−98.7 (6)
N1—Zn1—O1—C17	−66.7 (6)	N8—Zn2—N7—C27	8.1 (6)
O3—Na1—O2—C17	35.2 (10)	O4—Zn2—N7—C27	105.3 (6)
O10—Na1—O2—C17	133.4 (9)	O3—Zn2—N7—C27	162.5 (6)
O6—Na1—O2—C17	−63.7 (10)	N6—Zn2—N7—C24	32.9 (7)
O5—Na1—O2—C17	−120.0 (9)	N8—Zn2—N7—C24	139.7 (7)
O9—Na1—O2—C17	156.2 (9)	O4—Zn2—N7—C24	−123.1 (6)
O2—Na1—O3—C35	40.1 (9)	O3—Zn2—N7—C24	−65.9 (7)
O10—Na1—O3—C35	−58.2 (9)	N6—Zn2—N8—C28	112.4 (5)
O6—Na1—O3—C35	133.5 (9)	N5—Zn2—N8—C28	−162.1 (6)
O5—Na1—O3—C35	176.4 (8)	O4—Zn2—N8—C28	−62.3 (6)
O9—Na1—O3—C35	−112.0 (9)	N7—Zn2—N8—C28	22.3 (6)
O2—Na1—O3—Zn2	−154.1 (5)	O3—Zn2—N8—C28	−74.0 (9)
O10—Na1—O3—Zn2	107.6 (5)	N6—Zn2—N8—C30	−123.4 (6)
O6—Na1—O3—Zn2	−60.7 (6)	N5—Zn2—N8—C30	−37.9 (6)
O5—Na1—O3—Zn2	−17.8 (9)	O4—Zn2—N8—C30	61.9 (6)
O9—Na1—O3—Zn2	53.9 (5)	N7—Zn2—N8—C30	146.5 (6)
N6—Zn2—O3—C35	−171.8 (6)	O3—Zn2—N8—C30	50.2 (10)
N8—Zn2—O3—C35	14.3 (10)	C14—N1—C1—C2	−169.8 (8)
N5—Zn2—O3—C35	104.1 (6)	Zn1—N1—C1—C2	−40.2 (8)
O4—Zn2—O3—C35	0.7 (5)	C4—N2—C2—C1	−174.7 (7)
N7—Zn2—O3—C35	−78.5 (6)	Zn1—N2—C2—C1	−44.0 (8)
N6—Zn2—O3—Na1	18.8 (6)	N1—C1—C2—N2	60.3 (10)
N8—Zn2—O3—Na1	−155.1 (6)	C2—N2—C4—C3	−54.5 (11)
N5—Zn2—O3—Na1	−65.2 (5)	Zn1—N2—C4—C3	−178.0 (7)
O4—Zn2—O3—Na1	−168.7 (6)	C2—N2—C4—C5	−178.6 (8)
N7—Zn2—O3—Na1	112.2 (5)	Zn1—N2—C4—C5	57.8 (9)
N6—Zn2—O4—C35	17.6 (10)	N2—C4—C5—C6	−71.6 (10)
N8—Zn2—O4—C35	−174.9 (6)	C3—C4—C5—C6	163.4 (8)
N5—Zn2—O4—C35	−80.2 (6)	C9—N3—C6—C7	−45.6 (10)
N7—Zn2—O4—C35	102.5 (6)	Zn1—N3—C6—C7	−171.8 (6)
O3—Zn2—O4—C35	−0.7 (5)	C9—N3—C6—C8	−166.2 (8)
O7—Cl1—O5—Na1	−115.9 (7)	Zn1—N3—C6—C8	67.7 (8)
O8—Cl1—O5—Na1	123.7 (6)	C9—N3—C6—C5	75.1 (10)
O6—Cl1—O5—Na1	2.8 (6)	Zn1—N3—C6—C5	−51.1 (9)
O2—Na1—O5—Cl1	84.2 (5)	C4—C5—C6—C7	−171.3 (9)
O3—Na1—O5—Cl1	−55.7 (8)	C4—C5—C6—N3	67.0 (11)
O10—Na1—O5—Cl1	−178.2 (4)	C4—C5—C6—C8	−48.8 (11)
O6—Na1—O5—Cl1	−1.9 (4)	C6—N3—C9—C10	−173.6 (8)
O9—Na1—O5—Cl1	−129.0 (5)	Zn1—N3—C9—C10	−40.1 (8)
O7—Cl1—O6—Na1	114.5 (6)	C12—N4—C10—C9	−176.3 (8)
O8—Cl1—O6—Na1	−123.3 (6)	Zn1—N4—C10—C9	−45.3 (8)
O5—Cl1—O6—Na1	−2.9 (6)	N3—C9—C10—N4	60.6 (10)
O2—Na1—O6—Cl1	−95.1 (5)	C10—N4—C12—C13	179.0 (8)
O3—Na1—O6—Cl1	152.6 (5)	Zn1—N4—C12—C13	55.1 (10)
O10—Na1—O6—Cl1	13.6 (14)	C10—N4—C12—C11	−55.5 (11)
O5—Na1—O6—Cl1	2.0 (4)	Zn1—N4—C12—C11	−179.5 (6)
O9—Na1—O6—Cl1	62.2 (5)	N4—C12—C13—C14	−69.8 (13)
O11—Cl2—O9—Na1	−119.9 (5)	C11—C12—C13—C14	163.3 (10)
O12—Cl2—O9—Na1	118.1 (5)	C12—C13—C14—N1	66.4 (13)
O10—Cl2—O9—Na1	−2.5 (5)	C12—C13—C14—C15	−54.8 (13)
O2—Na1—O9—Cl2	−26.7 (7)	C12—C13—C14—C16	−173.6 (10)
O3—Na1—O9—Cl2	100.6 (4)	C1—N1—C14—C13	72.2 (10)
O10—Na1—O9—Cl2	1.8 (3)	Zn1—N1—C14—C13	−50.3 (9)
O6—Na1—O9—Cl2	−161.7 (3)	C1—N1—C14—C15	−163.4 (9)
O5—Na1—O9—Cl2	−114.7 (4)	Zn1—N1—C14—C15	74.1 (9)
O11—Cl2—O10—Na1	121.6 (5)	C1—N1—C14—C16	−46.1 (11)
O12—Cl2—O10—Na1	−119.3 (4)	Zn1—N1—C14—C16	−168.6 (6)
O9—Cl2—O10—Na1	2.8 (5)	Zn1—O1—C17—O2	−11.8 (10)
O2—Na1—O10—Cl2	163.1 (4)	Zn1—O1—C17—C18	167.9 (7)
O3—Na1—O10—Cl2	−84.1 (5)	Na1—O2—C17—O1	−177.0 (6)
O6—Na1—O10—Cl2	55.2 (12)	Na1—O2—C17—C18	3.2 (14)
O5—Na1—O10—Cl2	65.3 (5)	C32—N5—C19—C20	−171.2 (7)
O9—Na1—O10—Cl2	−1.8 (3)	Zn2—N5—C19—C20	−37.3 (8)
O1—Zn1—N1—C1	160.3 (5)	N5—C19—C20—N6	58.6 (10)
N4—Zn1—N1—C1	−93.2 (6)	C22—N6—C20—C19	−176.7 (8)
N2—Zn1—N1—C1	13.2 (6)	Zn2—N6—C20—C19	−46.3 (8)
O1—Zn1—N1—C14	−71.0 (6)	C20—N6—C22—C21	−57.1 (11)
N4—Zn1—N1—C14	35.5 (6)	Zn2—N6—C22—C21	−179.8 (7)
N2—Zn1—N1—C14	141.9 (6)	C20—N6—C22—C23	179.9 (8)
O1—Zn1—N2—C2	−77.7 (8)	Zn2—N6—C22—C23	57.2 (9)
N4—Zn1—N2—C2	107.1 (6)	N6—C22—C23—C24	−74.6 (11)
N3—Zn1—N2—C2	−168.1 (6)	C21—C22—C23—C24	161.3 (9)
N1—Zn1—N2—C2	16.1 (6)	C22—C23—C24—C25	−168.7 (8)
O1—Zn1—N2—C4	51.0 (9)	C22—C23—C24—C26	−48.7 (11)
N4—Zn1—N2—C4	−124.1 (6)	C22—C23—C24—N7	68.9 (10)
N3—Zn1—N2—C4	−39.3 (7)	C27—N7—C24—C25	−41.7 (11)
N1—Zn1—N2—C4	144.8 (7)	Zn2—N7—C24—C25	−168.4 (6)
O1—Zn1—N3—C9	119.1 (6)	C27—N7—C24—C23	79.3 (9)
N4—Zn1—N3—C9	12.8 (6)	Zn2—N7—C24—C23	−47.5 (9)
N2—Zn1—N3—C9	−93.7 (6)	C27—N7—C24—C26	−160.4 (8)
O1—Zn1—N3—C6	−110.2 (6)	Zn2—N7—C24—C26	72.8 (8)
N4—Zn1—N3—C6	143.5 (6)	C24—N7—C27—C28	−170.3 (8)
N2—Zn1—N3—C6	37.0 (6)	Zn2—N7—C27—C28	−36.6 (9)
O1—Zn1—N4—C12	59.3 (7)	C30—N8—C28—C27	−173.8 (8)
N2—Zn1—N4—C12	−123.4 (7)	Zn2—N8—C28—C27	−49.3 (8)
N3—Zn1—N4—C12	147.0 (7)	N7—C27—C28—N8	61.2 (10)
N1—Zn1—N4—C12	−37.8 (7)	C28—N8—C30—C29	−59.2 (11)
O1—Zn1—N4—C10	−71.0 (6)	Zn2—N8—C30—C29	−177.9 (7)
N2—Zn1—N4—C10	106.3 (6)	C28—N8—C30—C31	178.1 (8)
N3—Zn1—N4—C10	16.7 (5)	Zn2—N8—C30—C31	59.3 (9)
N1—Zn1—N4—C10	−168.1 (5)	N8—C30—C31—C32	−75.7 (11)
N6—Zn2—N5—C32	140.2 (7)	C29—C30—C31—C32	160.2 (9)
N8—Zn2—N5—C32	33.5 (7)	C19—N5—C32—C33	−159.6 (7)
O4—Zn2—N5—C32	−63.8 (7)	Zn2—N5—C32—C33	73.6 (8)
O3—Zn2—N5—C32	−121.0 (6)	C19—N5—C32—C34	−39.5 (11)
N6—Zn2—N5—C19	9.3 (6)	Zn2—N5—C32—C34	−166.2 (7)
N8—Zn2—N5—C19	−97.4 (6)	C19—N5—C32—C31	79.9 (9)
O4—Zn2—N5—C19	165.3 (5)	Zn2—N5—C32—C31	−46.9 (9)
O3—Zn2—N5—C19	108.1 (6)	C30—C31—C32—N5	67.2 (11)
N8—Zn2—N6—C22	−121.1 (6)	C30—C31—C32—C33	−51.3 (11)
N5—Zn2—N6—C22	146.8 (6)	C30—C31—C32—C34	−169.5 (8)
O4—Zn2—N6—C22	45.9 (10)	Zn2—O4—C35—O3	1.3 (10)
N7—Zn2—N6—C22	−37.6 (6)	Zn2—O4—C35—C36	−178.2 (8)
O3—Zn2—N6—C22	61.6 (6)	Na1—O3—C35—O4	169.9 (6)
N8—Zn2—N6—C20	111.3 (5)	Zn2—O3—C35—O4	−1.2 (9)
N5—Zn2—N6—C20	19.2 (5)	Na1—O3—C35—C36	−10.6 (14)
O4—Zn2—N6—C20	−81.6 (8)	Zn2—O3—C35—C36	178.3 (9)

### Hydrogen-bond geometry (Å, °)

**Table:**

*D*—H···*A*	*D*—H	H···*A*	*D*···*A*	*D*—H···*A*
N2—H2···O15^i^	0.88	2.43	3.27 (1)	160
N4—H4···O15^i^	0.88	2.44	3.24 (1)	152
N6—H6···O14^ii^	0.88	2.35	3.17 (1)	154
N8—H8···O15^ii^	0.88	2.46	3.29 (1)	158

Symmetry codes: (i) *x*, *y*, *z*+1; (ii) *x*, *y*−1, *z*.
